# Hair Follicle Morphogenesis During Embryogenesis, Neogenesis, and Organogenesis

**DOI:** 10.3389/fcell.2022.933370

**Published:** 2022-07-22

**Authors:** Sangbum Park

**Affiliations:** ^1^ Institute for Quantitative Health Science & Engineering (IQ), Michigan State University, East Lansing, MI, United States; ^2^ Division of Dermatology, Department of Medicine, College of Human Medicine, Michigan State University, East Lansing, MI, United States; ^3^ Department of Pharmacology and Toxicology, College of Human Medicine, Michigan State University, East Lansing, MI, United States

**Keywords:** hair follicle stem cells (HFSCs), stem cell niche, wound-induced hair neogenesis (WIHN), hair follicle organoid, hair follicle (HF)

## Abstract

Hair follicles are mini organs that repeat the growth and regression cycle continuously. These dynamic changes are driven by the regulation of stem cells via their multiple niche components. To build the complex structure of hair follicles and surrounding niches, sophisticated morphogenesis is required during embryonic development. This review will explore how hair follicles are formed and maintained through dynamic cellular changes and diverse signaling pathways. In addition, comparison of differences in stem cells and surrounding niche components during embryogenesis, neogenesis, and organogenesis will provide a comprehensive understanding of mechanisms for hair follicle generation and insights into skin regeneration.

## Introduction

The skin is the largest and outermost organ of our body. The major role of the skin is to protect our body from external insults, such as temperature changes, radiation, pathogens, and physical and chemical damages. The skin performs these barrier functions along with appendages including hair follicles, sebaceous glands, sweat glands, and nails. Among them, the hair follicle is the most studied appendage in the skin. Majority of the skin area has hair follicles except for palms, soles, and lips. Hair has various functions of protection. First, hair helps to control body temperature. Hair traps warm air on the skin surface and creates an insulating layer from the cold temperatures outside. Conversely, hair blocks direct sunlight on the skin surface and prevents the skin temperature from rising rapidly. Second, hairs protect our bodies from damage. Hair prevents dangerous substances from coming into direct contact with the skin and acts as a cushioning material from a physical strike. Third, hairs feel a sense of touch. Several mechanosensory receptors form specialized terminals by surrounding hair follicles in the dermis (Zimmerman et al., 2014). These sensory receptors enable the detection of movement of hair shafts and extend the sense of touch beyond the skin surface. Although hair plays such an important protective role, destroyed hair follicles cannot be repaired in adults. However, recently, several studies have reported methods for generating hair follicles in adult mice as well as in culture dishes. This review will compare hair follicle morphogenesis under different conditions with respect to morphology, signaling pathways, and surrounding niches for hair follicle stem cells (HFSCs). These comparisons from various angles will provide insights into hair follicle genesis and skin regeneration.

## Hair Follicle Morphogenesis During Embryonic Development

**FIGURE 1:**
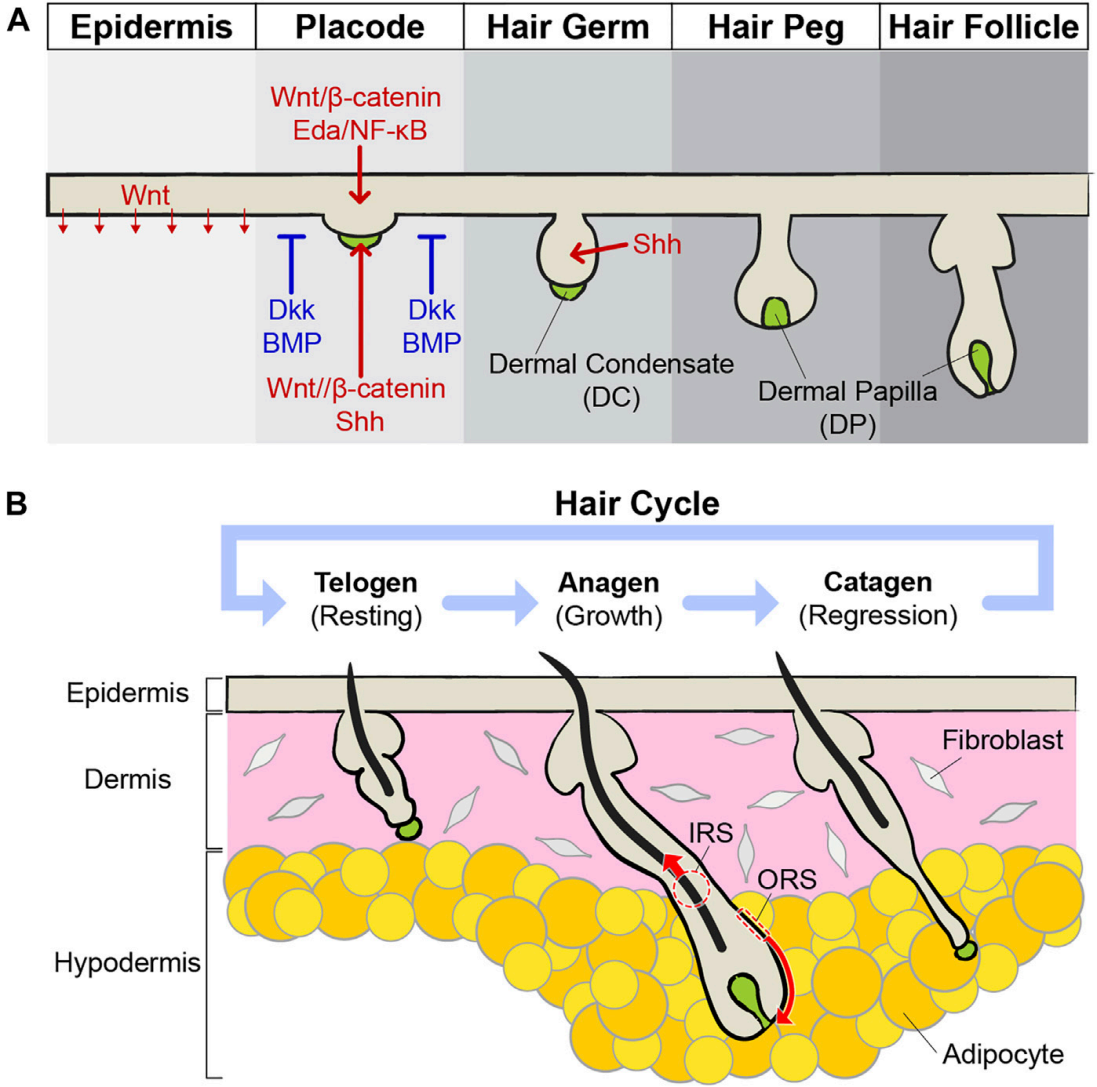
Development and cycling of hair follicles. **(A)** During embryonic development, the skin epithelium differentiates and generates hair follicles. Epithelial cells in the epidermis are thickening to build a placode, and mesenchymal cells in the dermis gather to form a dermal condensate (DC) just below the placode. The placode and DC exchange growth signals, such as Wnt/β-catenin and Shh, with each other and grow downward. Around the placodes, inhibitory signals, such as Dkk and BMP, suppress the expression of the hair follicles, thereby expressing the pattern of hair follicle arrays. Placode continuously develops into hair germ and hair peg structures. DC becomes dermal papilla (DP) right below the hair follicles and acts as niches for HFSCs. Eventually, mature hair follicles are formed prenatally. **(B)** Mature hair follicles undergo cycles of growth (anagen), regression (catagen), and resting (telogen). During the anagen, stem cells become activated by the surrounding niche components. Activated stem cells grow by repeating division, and their progenies differentiate to produce hair. When the anagen stops, the hair follicles enter the catagen phase. Through apoptosis of the outer root sheath (ORS) and extrusion of the inner root sheath (IRS), hair follicles become short in a few days. During the telogen, stem cells in the hair follicles are maintained in quiescence by inhibitory signals. When telogen is finished, stem cells are activated and the anagen starts again.

Morphogenesis of hair follicles has been well-characterized during embryonic development using mouse models (Xin et al., 2016; Paus et al., 1999). The hair follicle is composed of epithelial cells that are continuous with the interfollicular epidermis. Therefore, the morphogenesis of hair follicles occurs along with the development of the epidermis (Park, 2022). The epidermis originated from the surface ectoderm at embryonic day (E) 8.5 in mice and stratified into four different types of layers: basal, spinous, granular, and cornified layers, through differentiation during development (Koster and Roop, 2007). Cells in the upper dermis activate Wnt/β-catenin signaling broadly by receiving Wnt ligands from the epithelial cells at E12.5–14.5 for the hair follicle morphogenesis (Zhang et al., 2009; Chen et al., 2012). Epithelial cells, which receive the first signal from the dermis, have activated Wnt/β-catenin and ectodysplasin (Eda)/nuclear factor-κB (NF-κB) signaling for thickening of epithelial cells, known as placode, and they secrete fibroblast growth factor (FGF) 20 for the specification of dermal condensates (DCs), which is the clustering group of mesenchymal cells (Zhang et al., 2009; Mok et al., 2019). FGF20 is required to modulate the timing and level of Wnt and Sonic hedgehog (Shh) signaling which mediate DC specification. However, FGF20 is not absolutely required because DCs can be formed in FGF20 knockout, although these DCs are delayed and smaller (Qu et al., 2022). (Figure 1A). Surrounding interfollicular cells activate inhibitory signals, such as Dickkopf (Dkk) and bone morphogenetic protein (BMP), to block hair follicle formation (Mou et al., 2006; Sick et al., 2006; Gupta et al., 2019). These inhibitory signals determine the pattern of the hair follicle array (Xin et al., 2016). Live imaging of embryonic skin explants during the skin placode formation revealed that placode formation is driven by cell motility, such as intercalation, condensation, and directional migration, rather than proliferation. Activated Wnt/β-catenin and Eda/NF-κB signaling increase cell motility and suppress proliferation (Ahtiainen et al., 2014). In contrast to the placode, proliferation is necessary to generate dermal condensation. Recent single-cell RNA-seq analysis revealed that DC progenitors are initially highly proliferative. However, Shh signaling causes a rapid transition to quiescent and mature DC within a short time frame (Qu et al., 2022). Once the placode and DC are formed, the placode develops into hair germ and hair peg by growing downward, and DCs surrounded by epithelial cells develop into dermal papilla (DP) (Millar, 2002) (Figure 1A). Although Shh signaling does not impact hair morphogenesis until stage placode formation, activation of Shh signaling via interactions between placode and DC plays a critical role in growing placode to the hair peg (St-Jacques et al., 1998; Chiang et al., 1999; Ouspenskaia et al., 2016). Further epithelial–mesenchymal interactions lead to proliferation and differentiation of epithelial cells in the hair peg and to the fully matured hair follicle (Schneider et al., 2009) (Figure 1A).

From placode formation to mature hair follicle development, the skin epithelium underwent dramatic shape changes. However, it was still unclear how each cell in the placode changes its lineage during hair follicle morphogenesis. Recently, live imaging of *ex vivo* skin culture followed the development of embryonic whisker hair follicles up to 11 days (Morita et al., 2021). The long-term lineage tracing data revealed that cell fate is predetermined, based on initial position in the placode. Cells in the center become lower hair bulb cells and in the peripheral ring of the placode become future HFSCs in the hair bulge (Morita et al., 2021). These results revealed that the spatial arrangement is also an important factor in cell lineage and resembles adult hair follicle growth (Rompolas et al., 2013; Xin et al., 2018). Additional studies will be needed to interrogate further cellular mechanisms in hair follicle morphogenesis, driven by the spatial organization, such as the early formation of concentric ring structure and flexibility of cell fates like adult epithelial stem cells (Blanpain and Fuchs, 2014).

## Cycling of Adult Hair Follicles During Homeostasis

Mature hair follicles undergo growth cycles by interactions between stem cells and surrounding niches (Xin et al., 2016). The hair cycle has three phases: anagen, catagen, and telogen. The anagen is the growth phase (Figure 1B). During the anagen, the dermis and hypodermis become thicker and hair follicles grow down into the fat layer. To initiate hair follicle growth, interactions between the hair germ and DP are essential. The DP works as a signaling center for hair growth *via* Noggin and FGF7 (Greco et al., 2009; Hsu et al., 2011; Hsu et al., 2014). Depletion of DPs by laser ablation during the telogen blocks the hair follicles from entering the growth phase, and the hair follicles stay as telogen (Rompolas et al., 2012). Once hair follicle growth is initiated, cell division of hair follicle bulge stem cells and their progenies are dramatically increased. As the hair follicle grows, the hair germ surrounds the lower DP, and this process undergoes a dynamic structural change like embryonic morphogenesis. This morphological change is highly organized. The initial position of stem cells is predetermined where they are located after shape changes, and spatial location determines the fates of their progenies after differentiation eventually (Xin et al., 2018). As the hair follicle grows downward, the cells of the outer root sheath (ORS) are constantly dividing and moving downward. In addition, inner root sheath (IRS) cells, adjacent to the DP, generate a hair upwards through robust differentiation (Xin et al., 2018). Once the anagen is finished, hair growth stops and enters the catagen phase. The catagen is a regression phase and usually shorter than other phases (Figure 1B). IRS and ORS are removed in different ways. IRS cells are released upward like in the anagen phase, but ORS cells undergo apoptosis (Martino et al., 2021). The DP also plays an important role in the catagen. If the DP is removed, IRS cells are removed normally, but cell death of ORS cells is decreased. Therefore, hair follicles maintain long epithelial strands for a long time after DP ablation (Mesa et al., 2015). This niche-induced cell death is regulated by TGF-ß signaling from the DP during the early catagen (Foitzik et al., 2000). Once the catagen is finished, the hair follicles enter the telogen phase for resting (Figure 1B). HFSCs remain quiescent during the telogen and the DP contributes to this silent state of stem cells by regulating high BMP and low Wnt signaling (Quist and Quist, 2021). When telogen is finished, stem cells are activated and enter anagen again. Hair follicles repeat this growth/regression/resting cycle several times during their lifetime.

**FIGURE 2:**
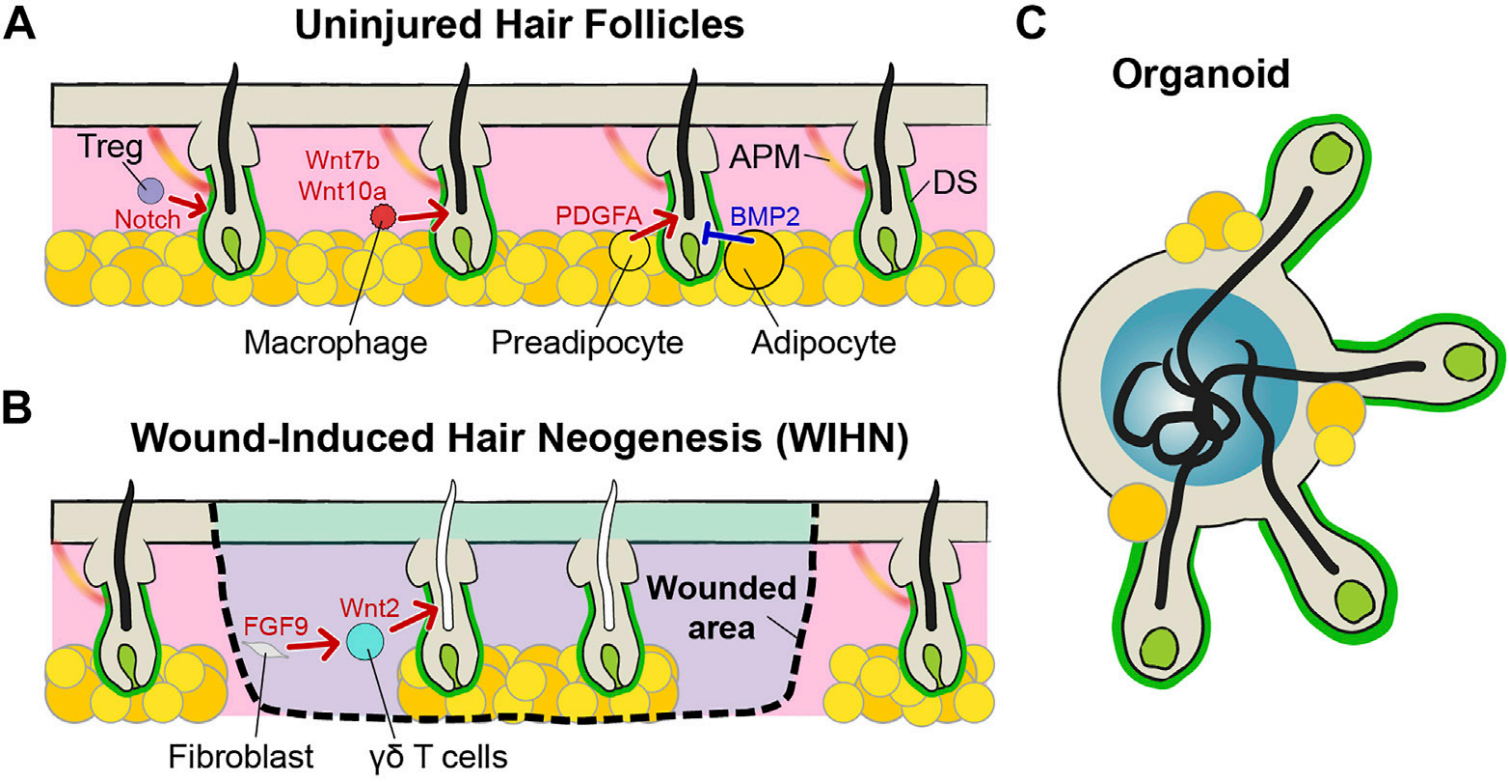
Niches of hair follicles. **(A)** Mature hair follicles have many niche components around them. In addition to DP, there are dermal sheath (DS) cells, regulatory T (Treg) cells, macrophages, preadipocytes, adipocytes, and arrector pili muscle (APM). These components of the niches regulate the homeostasis of hair follicles, cooperatively. **(B)** In terms of hair follicles from wound-induced hair neogenesis (WIHN), Wnt2 and FGF9 form a positive feedback loop and enhance the new hair generation. Most of the niches are composed, but melanocytes and APM are absent. Therefore, functional differences exist, such as being able to create only gray hairs. **(C)** In the case of hair follicles made by organoids, the structures of hair follicles and DPs are similar to those of general hair follicles. Due to the limitations of the organoid culture methods, circulatory systems, including blood and lymphatic vessels, do not exist. In addition, other cellular and non-cellular components are not perfect, such as immune cells and extracellular matrix (ECM). However, unlike the WIHN-derived hair follicles, melanocytes exist and hair follicles from the organoid can produce pigmented hairs.

In addition to the DP, additional niches surround hair follicles and regulate the homeostasis of hair follicles (Figure 2A). The dermal sheath (DS) is composed of mesenchymal cells surrounding hair follicles. DS cells are directly attached outside hair follicles and separated from ORS by the basement membrane (Martino et al., 2021). A lineage tracing study discovered that hair follicle dermal stem cells (hfDSCs) exist within the DS and self-renew. As hair cycles, the hfDSCs and the DP exchange their cell populations. Progenies of the hfDSCs enter the DP to contribute to the maintenance of the DP over cycling, and some progenies exit to the DS during catagen (Rahmani et al., 2014). These dynamic cellular exchanges cause fluctuation of DP cell numbers and eventually impact the hair type changes (Tobin et al., 2003; Rahmani et al., 2014). In addition to the DP regulation, DS cells also contribute catagen by providing contractile force, like smooth muscles. Intravital imaging of catagen hair follicles shows that contraction of the DS pushes IRS cells and hair shafts, like squeezing toothpaste. Blocking contraction abrogates upward movement of hair shafts (Heitman et al., 2020).

Immune cells also act as niches for HFSCs. Regulatory T (Treg) cells are generally well-known for their role in immune tolerance, but these cells also play an important role in the initiation of anagen (Sakaguchi et al., 2008). Activated forkhead box P3 (FOXP3)-expressing Treg cells are accumulated near the telogen follicles. These Treg cells control hair regeneration by activating the HFSCs via notch signaling and activated stem cells initiate new hair growth (Ali et al., 2017). Skin resident macrophages also impact HFSCs by regulating their number for hair cycling like the FOXP3-expressing Treg. Perifollicular macrophages decrease in number before anagen via apoptosis. Apoptotic macrophages activate HFSCs with Wnt7b and Wnt10a production and initiate anagen (Castellana et al., 2014).

Subcutaneous adipocytes are another niche component for hair follicles (Zwick et al., 2018). When the hair follicles begin the growth cycle, the adipose layer at the bottom also gets thicker (Rivera-Gonzalez et al., 2014). The growth of the adipose layer occurs when preadipocytes differentiate into mature adipocytes. These preadipocytes secrete platelet-derived growth factor subunit A (PDGFA), which activates HFSC to initiate anagen (Festa et al., 2011). In contrast, mature adipocytes maintain telogen by expressing BMP2 to keep HFSCs in quiescence (Plikus et al., 2008).

As mentioned previously, sensory nerves are wrapped around hair follicles. The sensory nerves not only perform mechanosensory functions but also regulate the fate of HFSCs. Innervation of sensory neurons maintains Gli1 or Lgr6 positive stem cells in the hair follicle by releasing Shh or by physically contacting them, respectively. These stem cells functionally contribute to re-epithelialization after skin injury (Brownell et al., 2011; Huang et al., 2021). Arrector pili muscle (APM) is a thin muscle that is responsible for piloerection when people are cold or scared (Fujiwara et al., 2011). The APM is directly in contact with the hair bulge because HFSCs create a niche for these muscle cells by expressing nephronectin (Fujiwara et al., 2011). In contrast, APM acts as a niche for HFSCs by maintaining sympathetic nerve innervation to stem cells. Through this connection, cold stimulates the activation of HFSCs and hair growth (Shwartz et al., 2020).

Lymphatic vessels have been recently identified as a niche component. Lymphatic capillaries are closely associated with HFSCs. During the telogen phase, adjacent lymphatics maintain the quiescence of stem cells. However, once anagen is initiated, the secretome from activated stem cells, such as Ntn4 and Angpt4, remodels the lymphatic niches by dissociation of lymphatics from the HFSCs and allows hair growth (Gur-Cohen et al., 2019).

In addition to these cellular niches, non-cellular components, such as hormones and extracellular matrix, also become part of the niches for stem cells (Fujiwara et al., 2011; Morgner et al., 2015; Choi et al., 2021; de Groot et al., 2021). Altogether, complex regulations between stem cells and various niches are essential to maintain the homeostasis of mature hair follicles in adults. Therefore, correct hair follicle development should be accompanied by the formation of proper niche components, not just simply forming hair follicles.

## Hair Follicle Neogenesis in Adult

Mammals have limited regeneration capacity as compared to regeneration of lower organisms, such as heart regeneration of zebrafish, limb regeneration of axolotl, and body regeneration of planarian (Mokalled and Poss, 2018). Although some species show dramatic regeneration capacity of the skin, such as African spiny mice, majority of mammals cannot fully regenerate skin to its original form that includes skin appendages (Seifert et al., 2012). After severe injuries, the skin forms a scar and loses normal skin architecture, including hair follicles and sweat glands (desJardins- Park et al., 2019). Therefore, healed skin cannot properly perform functions, like temperature control after wound healing (Lin et al., 2021). Hair follicle *de novo* generation in adult mammals has been rarely observed in rabbits and sheep, but underlying mechanisms were unknown because of the limitation of the tools and model system (Billingham and Russell, 1956; Brook et al., 1960). However, Ito *et al.* demonstrated hair follicle regeneration during wound repair in mouse models. They found that wound-induced hair neogenesis (WIHN) occurs at the center of large wounds (>1 cm^2^) (Figure 2B) (Ito et al., 2005; Ito et al., 2007), and not in small wounds. The WIHN recapitulates embryonic development of hair follicles, including formation of placode, hair germ, and DP, and shares the same signaling including Wnt/ß-catenin and Shh (Ito et al., 2007; Rognoni et al., 2016; Lim et al., 2018; Sun et al., 2020). Although the morphogenesis and signaling pathways of hair follicles are the same, their surrounding niche environments are different. The Wnt2 expression of fibroblasts is initiated by FGF9, secreted from dermal γδ T cells. Wnt2 and FGF9 form a positive feedback loop and enhance Wnt signaling activation (Gay et al., 2013). Therefore, a robust population of dermal γδ T cells is one of the reasons that mice can generate hair follicles after injury in contrast to humans. Interestingly, transient Wnt signaling activation is better for hair follicle neogenesis than continuous high Wnt until late wound healing (Gay et al., 2013). If the number of phagocytic macrophages is high, macrophages are phagocytizing dermal Wnt inhibitor secreted frizzled-related protein (SFRP) 4. Therefore, Wnt signaling is consistently high and the scar is formed in the wounded area rather than the hair follicles (Gay et al., 2020). The scar is formed by the excess fibrous connective tissue due to the abnormal proliferation of myofibroblasts during wound healing. Through lineage tracing experiments, it has been shown that a distinct fibroblast lineage (Engrailed-1 lineage-positive fibroblasts, EPFs) plays a major role in scar formation (Rinkevich et al., 2015). However, some Engrailed-1 lineage-negative fibroblasts (ENFs) also newly express Engrailed-1 during wound repair. This Engrailed-1 activation is triggered by the yes-associated protein (YAP) pathway, which is a well-known mechano-transduction signaling (Rinkevich et al., 2015; Mascharak et al., 2021). Treatment of verteporfin, a YAP inhibitor, inhibited Engrailed-1 activation in ENPs and effectively prevented scar formation. In addition, inhibition of YAP signaling also regenerates new hair follicle regeneration by activating Trps1, a Wnt pathway regulator (Mascharak et al., 2022). Surprisingly, the area of skin, where the hair follicles are newly formed, is completely regenerated up to the subcutaneous fat layer (Plikus et al., 2017). This is because high BMP signaling in the corresponding region makes myofibroblasts differentiate into adipocytes (Shook et al., 2020). All these studies suggest that the regeneration of new hair follicles is not just simply making new epithelial appendages, but the complete restoration of the surrounding niche components. Much investigation is still needed for perfect reproduction, including insufficient regeneration of melanocytes or arrector pili muscles (Figure 2B) (Wier and Garz a, 2019; Ankawa and Fuchs, 2022).

Although morphogenesis of hair follicles does not occur naturally without injury, experimental approaches can produce new hair follicles in adult mice. Several studies have demonstrated that transplantation of isolated epithelial stem cells and/or DP cells into nude mice can generate new hair follicles *in vivo* (Kishimoto et al., 2000; Blanpain et al., 2004; Ehama et al., 2007; Zhang et al., 2020). In addition to the isolated cells, implanted reprogrammed cells with induced pluripotent stem cells (iPSCs) also generate hair follicles in live mice (Veraitch et al., 2013). These transplantation experiments are basically methods of inducing the epithelial-mesenchymal interaction similar to the development of hair follicle placode by implanting primed cells into the skin of live mice. In most cases, immune rejection is avoided by implanting into immunodeficient nude mice. There is an additional way to induce new hair follicle generation without cell transplantation. Wnt and Shh signaling are one of the key pathways for hair follicle development. However, activation of these signaling in adult skin causes tumor formation (Gat et al., 1998; Kasper et al., 2011; Brown et al., 2017). A recent study, which combined genetic and pharmacological approaches, revealed that temporal activation of Shh signaling can generate new hair follicles in adult mice. Genetic deletion of Ptch1, the inhibitory receptor gene of Shh in both epithelial and stromal cells, generates basal cell carcinoma (BCC)–like tumor growth as expected. However, subsequent Shh pathway inhibitor, vismodegib, and treatment restricted the tumor growth and kept the intact structure of hair follicles (Sun et al., 2020). Transplantation and signaling activation experiments suggest that adult skin may already have an environment for hair follicle morphogenesis. If epithelial and stromal cells can be properly activated, it is possible to regenerate hair follicles even in adult animals. However, there are still many obstacles to applying this approach to humans, such as tumor formation. If the appropriate signal can be controlled spatially and temporally, it will be possible to induce hair regeneration in humans without hair implants.

## Hair Follicle Organoid

Many technological advances have been made since Howard Green succeeded in culturing skin epithelial stem cells *in vitro* (Rheinwald and Green, 1975). A 3D culture system, based on the growth of stratified squamous epithelium grown at an air-liquid interface enables the formation of the same structure of epidermis in culture dishes (Carlson et al., 2008). However, the development of complex appendage structures, such as hair follicles and sweat glands, has not been achieved for a long time. Due to the rapid progress in an organoid culture system, various types of tissues can be made *in vitro* while maintaining structures similar to real tissue (Hofer and Lutolf, 2021). Hair follicle organoids are also successfully made with mouse and human-derived induced pluripotent stem cells (Figure 2C) (Lee et al., 2018; Lee et al., 2020). The hair follicle organoids mimic the actual hair follicle development process and matured hair follicles in the organoid can make the hair shafts functional (Lee et al., 2022). Interestingly, in a spherical organoid, hair follicles grow outward and hair shafts grow inward. As a result, hair shafts and cornified cells that have been shed are accumulated at the core of the organoid (Lee and Koehler, 2021). Unlike the WIHN, melanocytes are present and can produce pigmented hair. Although adipocytes, sensory neurons, and Schwann cells are present, the organoids still lack other cell populations, including sweat glands, blood vessels, arrector pili muscle (rarely observed), and immune cells (Lee et al., 2022). Therefore, advances in protocols will be needed to generate fully mature skin organoids comprising entire niche components.

## Conclusion

Tissue-specific stem cells are responsible for regeneration during adulthood. One of the most important functions of regeneration is to maintain and repair the intact function and structure of the tissue. However, humans have limited regenerative ability after birth, and this may be to prevent the occurrence of tumor formation due to excessive regeneration. In the case of the adult skin, injuries can cause irreversible tissue damages, such as scar formation and loss of skin appendages. However, recent studies have demonstrated the mechanisms that reduce scar formation and regenerate hair follicles through interactions between stem cells and their surrounding niches. Although there are still difficulties in the full regeneration of skin like sweat glands, an advanced understanding of adult stem cells and niches will provide a better direction for skin regeneration in the future.
